# Supplementary material for: Matching sensor ontologies through siamese neural networks without using reference alignment
Source: PeerJ Comput Sci. 2021 Jun 18;7:e602. doi: 10.7717/peerj-cs.602 (PMC8237319; doi:10.7717/peerj-cs.602)
Supplement: Supplemental Information 1 [file peerj-cs-07-602-s001.zip › 302/refalign.html~]

# (level 0) Alignment

## Source: http://oaei.ontologymatching.org/2009/benchmarks/101/onto.rdf

## Target: http://oaei.ontologymatching.org/2009/benchmarks/302/onto.rdf

## Correspondences

type = type
:   1.0

proceedings = booktitle
:   1.0

volume = volume
:   1.0

Unpublished < Resource
:   1.0

address = address
:   1.0

chapter = chapter
:   1.0

Chapter = InBook
:   1.0

editor = editor
:   1.0

InBook < InBook
:   1.0

series = series
:   1.0

date = publishedOn
:   1.0

title = title
:   1.0

Booklet < Publication
:   1.0

LectureNotes < Publication
:   1.0

url = softCopyURI
:   1.0

MastersThesis = MastersThesis
:   1.0

pages = pages
:   1.0

TechReport = TechReport
:   1.0

edition = edition
:   1.0

institution = institution
:   1.0

isPartOf = booktitle
:   1.0

organization = organization
:   1.0

publisher = publisher
:   1.0

school = school
:   1.0

Misc = Misc
:   1.0

Collection < Book
:   1.0

abstract = abstract
:   1.0

Academic < Publication
:   1.0

Article = Article
:   1.0

Informal < Publication
:   1.0

Book = Book
:   1.0

book = booktitle
:   1.0

PhdThesis = PhdThesis
:   1.0

Proceedings = Proceedings
:   1.0

number = number
:   1.0

Reference = Resource
:   1.0

InCollection = InCollection
:   1.0

author = author
:   1.0

Report < Publication
:   1.0

note = note
:   1.0

Part < Publication
:   1.0

Manual < Publication
:   1.0

InProceedings = InProceedings
:   1.0

journal = journal
:   1.0

Monograph < Book
:   1.0

keywords = keyword
:   1.0

Deliverable < Publication
:   1.0

size = softCopySize
:   1.0
